# Evaluation of qPCR reference genes in GH-overexpressing transgenic zebrafish (*Danio rerio*)

**DOI:** 10.1038/s41598-020-69423-y

**Published:** 2020-07-29

**Authors:** Gabriela T. Rassier, Tony L. R. Silveira, Mariana H. Remião, Larissa O. Daneluz, Amanda W. S. Martins, Eduardo N. Dellagostin, Hadassa G. Ortiz, William B. Domingues, Eliza R. Komninou, Mateus T. Kütter, Luis F. F. Marins, Vinicius Farias Campos

**Affiliations:** 1 Laboratório de Genômica Estrutural, Programa de Pós-Graduação em Bioquímica e Bioprospecção, Centro de Ciências Químicas, Farmacológicas e de Alimentos, Universidade Federal de Pelotas, Campus Universitário s/n, Pelotas, RS Cep: 96010-900 Brazil; 2 Laboratório de Biologia Molecular, Instituto de Ciências Biológicas, Universidade Federal do Rio Grande, Rio Grande, RS Brazil; 3 Laboratório de Genômica Estrutural, Programa de Pós-Graduação em Biotecnologia, Centro de Desenvolvimento Tecnológico, Universidade Federal de Pelotas, Pelotas, RS Brazil; 4 Laboratório de Reprodução Animal, Programa de Pós-Graduação em Veterinária, Universidade Federal de Pelotas, Pelotas, RS Brazil

**Keywords:** Reverse transcription polymerase chain reaction, Gene expression analysis, Genetic engineering

## Abstract

Reference genes (RGs) must have a stable expression in tissues in all experimental conditions to normalize real-time quantitative reverse transcription PCR (qRT-PCR) data. F0104 is a highly studied lineage of zebrafish developed to overexpress the growth hormone (GH). It is assumed that the transgenic process may influence the expression levels of commonly used RGs. The objective of the present study was to make a comprehensive analysis of stability of canditade RGs *actb1, actb2, b2m, eif2s2, eef1a1, gapdh, rplp2, rpl7, rpl13α, tuba1,* and *rps18,* in *gh*-transgenic and non-transgenic zebrafish. Liver, brain, intestine and muscle samples from both groups had qRT-PCR results analyzed by dCt, geNorm, NormFinder, BestKeeper, and RefFinder softwares. Consensus analyses among software concluded that *rpl13α*, *rpl7*, and *eef1a1* are the most stable genes for zebrafish, considering the studied groups and tissues. *Gapdh*, *rps18*, and *tuba1* suffered variations in stability among different tissues of both groups, and so, they were listed as the genes with lowest stability. Results from an average pairwise variations test indicated that the use of two RGs would generate reliable results for gene expression analysis in the studied tissues. We conclude that genes that are commonly used in mammals for qRT-PCR assays have low stability in both non-transgenic and gh-transgenic zebrafish reinforcing the importance of using species-specific RGs.

## Introduction

Gene expression analysis using real-time quantitative reverse transcription polymerase chain reaction (qRT-PCR) is widely used for amplification, detection, and quantification of RNA in different types of biological animal samples^1, 2^. However, for the normalization and adequate reading of the obtained data, it is necessary to use reference genes (RGs) that must keep their expression stable, regardless of the conditions or stage of development, remaining constant in different tissues or under the influence of different experimental treatments^2–4^.

The indiscriminate use of RGs that have been confirmed for mammalian species to normalize the relative quantification in qRT-PCR for non-mammalian species is a common fact. Some of these genes are unsuitable to normalize data in fish species and could lead to a misinterpretation of results^4^. There are evidences that even described RGs can be regulated differently according to the conditions. That is the reason for validation of different RGs for each species and their developmental stages, tissues, as well as environmental conditions^4–6^.

The zebrafish (*Danio rerio*) is a model organism of increasing importance for developmental analyses, disease modeling and toxicological studies^7–9^. RGs have already been evaluated in wild zebrafish across developmental stages and considering differences of gender, tissues, chemical treatments and experimental protocols^1, 10–16^. The genetic background of the animals is a factor that can affect the differential expression of various genes, including RGs^17^. Thus, transgenic lineages must have a panel of RGs evaluated, independently of evaluation performed in the wild animals.

The F0104 lineage is the first transgenic fish lineage from Brazil^17^. The animals from this lineage are zebrafish alternative models that overexpresses the growth hormone (GH) from the silverside (*Odontesthes argentinensis*). The manipulation of the *gh* gene has shown promising results regarding growth in fish; this lineage has shown heterogeneous growth in males, as well as probable early sexual maturation^18^, reduced capacity of the antioxidant system^19^, a significant decrease in the sperm quality parameters^20^, and a decrease in the maturation of the immune system^21^. As seen, the transgenesis of only *gh* is able to alter the pattern of genes expression of various physiological systems and this also reinforces the importance of researching RGs which are stable under these altered and unstable genomic conditions.

In this sense, the F0104 lineage has diverse physiological alterations triggered by the transgenesis. The hormonal and gene expression alterations already described in this lineage can make the systemic environment very unusual and some RGs can behave differently from common. Thus, we would like to verify if commonly used RGs are also suitable for this transgenic model. The present study aimed to verify the stability of 11 candidate RGs, indicating the most adequate and the most inadequate for normalizing gene expression analysis in qRT-PCR for *gh*-transgenic zebrafish.

## Materials and methods

### Animals and conditions

The transgenic zebrafish overexpressing GH used in this study were generated by Figueiredo and collaborators^17^. Transgenic and non-transgenic zebrafish were obtained by the crossbreeding between F0104 lineage and wild zebrafish. In addition to *gh* transgene, the *gh*-transgenic zebrafish expresses the green fluorescent protein (GFP) as a transgenic label. The non-transgenic fish were siblings of the transgenic zebrafish which did not incorporate the genetic construct into their genome. This study was conducted in compliance with institutional, national, or international guidelines for using animals and all the protocols used were approved by the Ethics Committee of the Federal University of Rio Grande (FURG), Brazil, under the code 23116.008403/2018-32.

*Gh*-transgenic and non-transgenic zebrafish groups were kept in a recirculating aquiculture system. A total of 42 zebrafish of both sexes (21 transgenic and 21 non-transgenic) were raised in six tanks containing 15 L of water (three tanks/group and seven animals/tank) from juvenile (1-month-old) until adult stage (11-month-old). Each tank was cleaned daily by sliding the bottom to remove any residue. The tanks were connected to a filter tank filled with zeolite, bio ceramic, an ultraviolet (UV) lamp, and a heater thermostat. The fish were fed twice a day (5 days a week) until apparent satiety. The diet was commercial food (Tetra ColorBits, Germany). The photoperiod was adjusted to 14:10 h, light/dark period. Management and maintenance of zebrafish were in compliance with the Zebrafish Book (https://www.zfin.org). During the breeding period, the water temperature was maintained at 26.4 ± 0.3 °C. The water pH (7.6 ± 0.1) and dissolved oxygen (> 6 mg L^−1^) parameters were measured daily in all tanks. Chemical parameters were measured once a week using commercial kits (Labcon Ammonia Alcon Fresh Water; Labcon Test Nitrite Alcon, Brazil). All water parameters were maintained according to the requirements for zebrafish, as reported by Reed and Jennings (2010)^22^.

### Tissue collection

The animals were euthanized using overdose of tricaine methanesulfonate (MS-222, Sigma-Aldrich, USA) at 400 mg L^−1^. The animals were weighed and measured. Samples of liver, brain, intestine, and caudal muscle of adult zebrafish were collected from both groups. The samples collected from 3 animals were mixed to compose a tissue pool, totalizing seven replicate tissue pools (from liver, brain, intestine, and muscle) for each experimental group. After the collection, samples were stored in liquid nitrogen (N_2_) until RNA extraction.

### RNA extraction and cDNA synthesis

RNA extraction and cDNA synthesis were performed according to Silveira and collaborators^4^. Briefly, total RNA was extracted from samples using TRIZOL Reagent (Thermo Fisher Scientific, USA), following the manufacturer's instructions. RNA was treated with the DNAse-free kit (Ambion, USA) to remove genomic DNA contamination. Subsequently, RNA concentration and purity were measured using a NanoVue Plus spectrophotometer (GE Healthcare Life Science, USA) and after, the samples were stored at − 80 °C. Complementary DNA (cDNA) was obtained using High Capacity cDNA Reverse Transcription (Applied Biosystems, USA) according to the manufacturer's recommendation at a concentration of 500 ng. Finally, cDNA was stored at − 20 °C until use.

### Gene expression analysis by qRT-PCR

The primers used in this study are presented in Table 1. The qRT-PCR was performed using the SYBR Green PCR Master Mix (Applied Biosystems, USA), and carried out in the Applied Biosystems 7500 Real-Time PCR System (Applied Biosystems, EUA). The amplification conditions were 95 °C for 10 min, 40 cycles at 95 °C for 15 s, and 60 °C for 1 min, followed by the conditions necessary for the calculation of the melting curve. The study used primers that have been validated for zebrafish (Table 1) for 11 candidate RGs (*actb1, actb2, b2m, eef1a1, eif2s2, gapdh, rpl13α, rpl7, rplp2, rps18,* and *tuba1*) belonging to different physiological pathways and representing different functions to evaluate expression stability. All reactions were performed in duplicate.

**Table 1:** Summary of candidate reference genes evaluated in the present study and primer sequences used for real-time PCR.

Gene name	Gene symbol	Function	Primer Sequences (5′ → 3′)	GenBank accession no	References
18s ribosomal RNA	*rps18*	Ribosomal subunit	F: TGCATGGCCGTTCTTAGTTG R: AGTCTCGTTCGTTATCGGAATGA	FJ915075	^4^
Glyceraldehyde-3-phosphate dehydrogenase	*gapdh*	Glycolytic enzyme	F: GATGGTCATGCAATCACAGTCTA R: ATCATACTTGGCAGGTTTCTCAA	BC095386	^15^
Beta-2-microglobulin	*b2m*	Beta chain of major histocompatibility	F: GCCTCCACCCCAGAGAAAGG R: GCGGTTTTTATTTACATGTTG	BC062841	^4^
Eukaryotic translation elongation factor 1 alpha 1	*eef1a1*	Factor for protein translation	F: GGGCAAGGGCTCCTTCAA R: CGCTCGGCCTTCAGTTTG	NM_131263	^20^
Ribosomal protein L13 alpha	*rpl13α*	60S ribosomal protein	F: TCTGGAGGACTGTAAGAGGTATGC R: AGACGCACAATCTTGAGAGCA	NM 212784	^15^
Actin, beta 1	*actb1*	Cytoskeletal protein	F: GCTGTTTTCCCCTCCATTGTT R: TCCCATGCCAACCATCACT	NM_131031	^4^
Actin, beta 2	*actb2*	Cytoskeletal protein	F: TGACCGAGCGTGGCTGCTACA R: CTTGATGTCACGGACAATTTCTCT	NM_181601	*
Ribosomal protein L7	*rpl7*	60S ribosomal protein	F: CAGAGGTATCAATGGTGTCAGCCC R: TTCGGAGCATGTTGATGGAGGC	NM 213644	^15^
Ribosomal protein, large P2	*rplp2*	60S ribosomal protein	F: GCCAAAGCCCATGTCTTCA R: GGGATCGAGGCTGATGATGA	NM 212743	^15^
Eukaryotic translation initiation factor 2, subunit 2 beta	*eif2s2*	Factor for initiation of protein synthesis	F: GAAAGCCAACAAGTAGAAGCAAA R: ACCCTGTTCAAAAGCTCATCATA	NM_212675	*
Tubulin, alpha 1	*tuba1*	Cytoskeletal protein	F: GAGACCGGAGCTGGAAAACA R: GGAAACCCTGGAGACCTGTG	NM 194388	^15^

*Primers designed for the present study.

### Data analysis

The normality distribution of biometric data was evaluated by the Shapiro–Wilk test. The unpaired Student’s *t* test (one-tailed) was used to compare the mean values between transgenic and non-transgenic groups. The obtained values were expressed as g ± standard error of the mean (SEM) and mm ± SEM for length and mass, respectively. Analyses of gene expression stability were performed using the comparative method delta-Ct (dCt)^23^ and geNorm^24^, NormFinder^25^, BestKeeper^26^, and RefFinder statistical approaches as previously reported^27, 28^. The results were expressed as mean ± standard deviation (SD) for the dCt method; average expression stability value for GeNorm and NormFinder; Pearson correlation coefficient (r) for the BestKeeper; and geometric mean of rating values for RefFinder.

Average pairwise variations (V) were calculated by geNorm between the normalization factors NFn and NFn + 1 to indicate whether inclusion of an extra RG would add to the stability of the normalization factor. The seven best RG candidates out of the eleven evaluated were used to calculate the V values. The use of seven genes was, independently of the results, due the inclusion of unstable genes (represented by the four worst genes that were excluded) which would be worse than the use of a few suitable RG.

### Verification

A protocol was established to demonstrate the differences found in gene expression stability between RGs by algorithms. The number of RGs used as ‘the best’ was determined by the pairwise variation analysis (the result was that two genes were necessary to normalize reactions efficiently, see in results section). The worst and the third best RG candidates of each tissue were used as target genes in gene expression analysis. Thus, the comparison of the implications of the use of unsuitable and suitable RGs in experimental analysis was possible. This procedure was performed in two parallel ways. In one way the qRT-PCR reactions were normalized with the two bests RGs of each tissue. In other way the reactions were normalized by the two bests RGs, considering all tissues grouped. This made it possible to compare if there are differences between the use of a single set of RGs for all tissues and the use of various sets of RGs, one set for each tissue. Because the third best liver RG was already committed with the normalization of ‘the first way’, this gene was replaced by the fourth best RG. Furthermore, this fact precluded ‘the second way’ of analysis because a redundancy would originate, since the same gene would be used as target gene and RG. The qRT-PCR data from the target genes were analyzed using the 2^−ΔΔCt^ method, according to Livak and Schmittgen (2001)^5^. The mean between the Cts of the RGs that were used was applied to the Livak and Schmittgen method. The normality distribution of the data was evaluated by the Shapiro–Wilk test. The unpaired Mann–Whitney test (two-tailed) was used to compare the mean values. The results were expressed as mean ± SD.

### Ethics approval

All the protocols used in this study were approved by the Ethics Committee of the Federal University of Rio Grande (FURG), Brazil, under the code 23116.008403/2018-32.

## Results

### Transgenic phenotype expression

**Figure 1:**
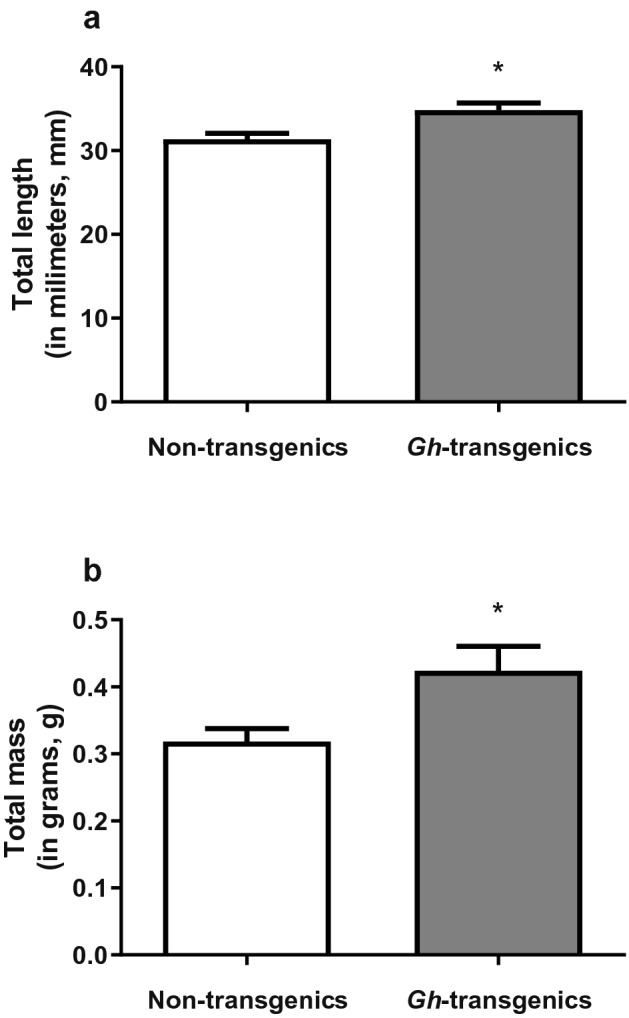
Biometry of non-transgenic and *gh*-transgenic zebrafish (*Danio rerio*) of F0104 lineage. Total length (**a**) and total mass (**b**) in non-transgenic zebrafish and in *gh*-transgenic zebrafish. Data are expressed as mean ± standard error of the mean. Asterisk indicate significant difference mean values between groups (n = 21; p < 0.05).

The transgenic group expressed the phenotype attributed by the transgenesis, being statistically longer and heavier (p < 0.05) (Fig. 1a,b, respectively) in comparison to the length and body mass of the non-transgenic group. The non-transgenic group presented 31.35 ± 0.68 mm and the transgenic group presented 34.83 ± 0.85 mm of total length. Furthermore, the non-transgenic group presented 0.31 ± 0.02 g and the transgenic group presented 0.42 ± 0.03 g of total mass.

### RefFinder analysis

**Figure 2:**
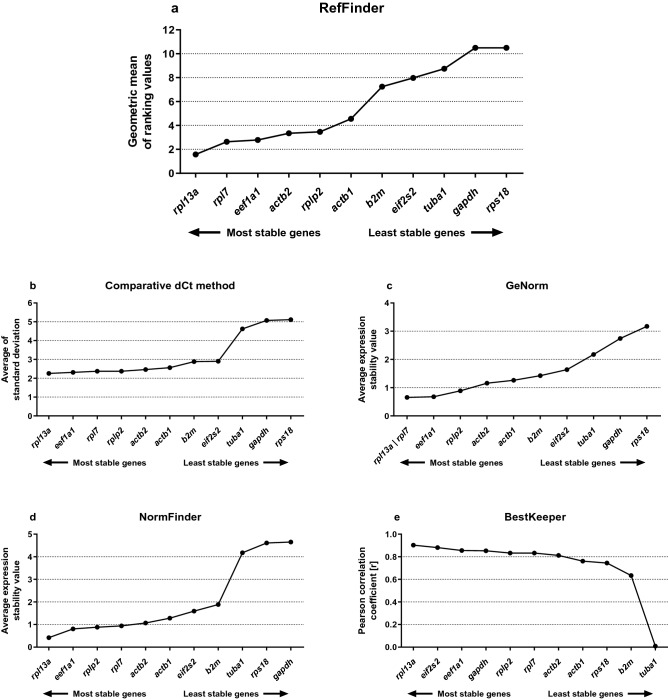
Consensus stability analyses of the candidates reference genes (RGs) grouping non-transgenic and *gh*-transgenic groups of zebrafish (*Danio rerio*) of F0104 lineage. Final consensus stability analysis between all methods calculated by RefFinder algorithm (**a**). Consensus stability analysis of the candidates RGs grouping both the groups calculed by comparative delta Ct (dCt) method (**b**); and geNorm (**c**); NormFinder (**d**); and BestKeeper (**e**) algorithms.

The RefFinder is a tool used to construct a consensus comprehensive ranking of stability of RGs among comparative dCt method and the geNorm, NormFinder, and BestKeeper algorithms using the calculation of geometric mean for the ranks calculated by each of these other methods. Candidate genes with the lowest geometric mean are most stable. A consensus graph resulted from RefFinder analysis considering all the analyzed methods, tissues, and combining both non-transgenic and *gh*-transgenic groups was generated and is presented together with the results of the other methods that supported the consensus analysis (Fig. 2a–e). This general consensus analysis revealed the follow ranking order: *rpl13a* (1.57) > *rpl7* (2.63) > *eef1a1* (2.78) > *actb2* (3.34) > *rplp2* (3.46) > *actb1* (4.56) > *b2m* (7.24) > *eif2s2* (7.97) > *tuba1* (8.74) > *gapdh* (10.49) = *rps18* (10.49) (Fig. 2a).

**Figure 3:**
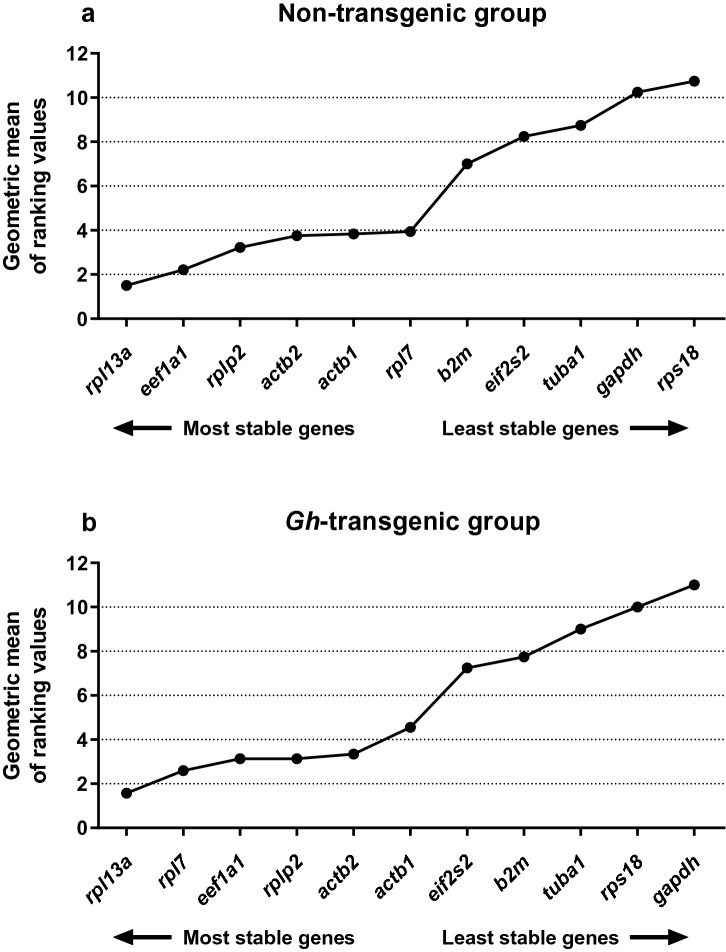
Consensus stability analysis in non-transgenic and *gh-*transgenic zebrafish (*Danio rerio*) of F0104 lineage calculated by RefFinder algorithm. General gene expression stability in non-transgenic zebrafish (**a**) and in *gh*-transgenic zebrafish (**b**). Data are expressed as geometric mean of ranking values. The most stable genes are displayed on the left, and the least stable genes are displayed on the right of the X-axis.

Furthermore, consensus results of RefFinder, individually discriminated by non-transgenic and *gh*-transgenic groups but combining all tissues, are also shown (Fig. 3a,b). In the non-transgenic group, the comprehensive consensus classification constructed by RefFinder (Fig. 3a) was: *rpl13a* (1.50) > *eef1a1* (2.21) > *rplp2* (3.22) > *actb2* (3.76) > *actb1* (3.83) > *rpl7* (3.94) > *b2m* (7.00) > *eif2s2* (8.24) > *tuba1* (8.74) > *gapdh* (10.24) > *rps18* (10.74). In the *gh*-transgenic group the classification order (Fig. 3b) was: *rpl13a* (1.57) > *rpl7* (2.59) > *eef1a1* (3.13) > *rplp2* (3.13) > *actb2* (3.34) > *actb1* (4.56) > *eif2s2* (7.24) > *b2m* (7.74) > *tuba1* (9.00) > *rps18* (10.00) > *gapdh* (11.00).

Finally, in addition to graphic rankings, RefFinder consensus results are presented for each group and by stability in each tissue (liver, brain, intestine, and muscle) (Tables 2, 3, 4). According to the consensus analysis of the tissues of both groups together, the most stable gene was considered the *rpl13α* for brain and intestine, occupying the second and third place for muscle and liver, respectively (Table 2). Also, in consensus analysis, the *gapdh* gene was the most unstable and unsuitable gene, except for the intestine, when *gapdh* was the second most unstable (Table 2).

**Table 2:** Comprehensive consensual stability ranking of the candidate reference genes in both non-transgenic and *gh*-transgenic zebrafish (*Danio rerio*) of F0104 lineage across the different tissues calculated by RefFinder algorithm. Data are expressed as geometric mean of ranking values.

Consensus between the groups		Tissues							
		Brain		Intestine		Liver		Muscle	
Ranking	1	*rpl13a*	1.78	*rpl13a*	2.06	*rpl7*	2.00	*actb2*	1.63
	2	*eef1a1*	2.45	*eef1a1*	2.34	*eef1a1*	2.14	*rpl13a*	2.06
	3	*b2m*	2.99	*actb1*	2.34	*rpl13a*	3.22	*actb1*	2.63
	4	*rpl7*	3.98	*actb2*	2.63	*rplp2*	4.16	*eef1a1*	4.12
	5	*rplp2*	4.16	*rpl7*	4.90	*b2m*	4.47	*rpl7*	4.70
	6	*actb1*	4.30	*b2m*	5.14	*actb2*	4.74	*tuba1*	4.95
	7	*actb2*	4.56	*rplp2*	5.86	*actb1*	4.76	*rplp2*	5.38
	8	*eif2s2*	8.00	*tuba1*	7.74	*eif2s2*	7.36	*b2m*	8.13
	9	*rps18*	9.24	*eif2s2*	9.00	*tuba1*	7.95	*eif2s2*	8.24
	10	*tuba1*	9.74	*gapdh*	10.00	*rps18*	9.00	*rps18*	10.00
	11	*gapdh*	11.00	*rps18*	11.00	*gapdh*	11.00	*gapdh*	11.00

In the non-transgenic group (Table 3), the best specific RG for brain and liver was *actb1*. The most stable genes in intestine and muscle were *rpl13a* and *actb2*, respectively. The worst genes found were *rps18* in brain and muscle and *gapdh* in intestine and liver. In the *gh*-transgenic group (Table 4), the most stable genes were the *actb1* in intestine and muscle; *eef1a1* in the brain; and *rplp2* in the liver. In this group, *rps18* was the most inappropriate gene for intestine and liver, while for brain and muscle were *eif2s2* and *b2m,* respectively (Table 4).

**Table 3:** Comprehensive consensual stability ranking of the candidate reference genes in non-transgenic zebrafish (*Danio rerio*) across the different tissues calculated by RefFinder algorithm. Data are expressed as geometric mean of ranking values.

Non-transgenic group		Tissues							
		Brain		Intestine		Liver		Muscle	
Ranking	1	*actb1*	2.11	*rpl13a*	1.78	*actb1*	1.19	*actb2*	2.43
	2	*rplp2*	2.21	*rpl7*	2.28	*actb2*	1.86	*rpl13a*	2.45
	3	*eef1a1*	2.21	*b2m*	2.78	*rpl7*	2.83	*b2m*	2.74
	4	*rpl13a*	3.20	*tuba1*	3.13	*rpl13a*	3.83	*eef1a1*	3.50
	5	*actb2*	3.94	*eef1a1*	3.74	*eef1a1*	4.61	*rplp2*	4.12
	6	*rpl7*	5.83	*actb2*	5.69	*b2m*	6.16	*actb1*	4.90
	7	*gapdh*	6.19	*actb1*	6.70	*rplp2*	6.44	*tuba1*	5.73
	8	*b2m*	8.21	*rplp2*	6.93	*eif2s2*	7.74	*rpl7*	5.80
	9	*tuba1*	8.80	*eif2s2*	8.74	*tuba1*	9.00	*eif2s2*	7.97
	10	*eif2s2*	8.97	*rps18*	10.00	*rps18*	10.24	*gapdh*	10.00
	11	*rps18*	11.00	*gapdh*	11.00	*gapdh*	10.74	*rps18*	11.00

**Table 4:** Comprehensive consensual stability ranking of the candidate reference genes in *gh*-transgenic zebrafish (*Danio rerio*) of F0104 lineage across the different tissues calculated by RefFinder algorithm. Data are expressed as geometric mean of ranking values.

*Gh*-transgenic group		Tissues							
		Brain		Intestine		Liver		Muscle	
Ranking	1	*eef1a1*	1.41	*actb1*	1.32	*rplp2*	1.86	*actb1*	1.73
	2	*rpl13a*	1.86	*actb2*	2.11	*eef1a1*	2.65	*actb2*	2.63
	3	*actb2*	2.71	*eef1a1*	2.21	*actb1*	3.22	*rpl7*	3.13
	4	*actb1*	3.31	*rpl13a*	3.83	*rpl7*	3.66	*eef1a1*	3.16
	5	*b2m*	4.47	*rplp2*	5.14	*b2m*	3.76	*rpl13a*	5.18
	6	*rpl7*	5.96	*rpl7*	5.38	*rpl13a*	3.94	*gapdh*	5.62
	7	*rplp2*	7.44	*b2m*	6.96	*actb2*	4.92	*rplp2*	6.00
	8	*tuba1*	7.45	*tuba1*	7.44	*gapdh*	8.00	*tuba1*	6.34
	9	*gapdh*	9.24	*eif2s2*	9.00	*tuba1*	9.24	*eif2s2*	7.71
	10	*rps18*	10.16	*gapdh*	10.00	*eif2s2*	9.74	*rps18*	7.91
	11	*eif2s2*	10.24	*rps18*	11.00	*rps18*	11.00	*b2m*	11.00

### The comparative dCt method analysis

The comparative dCt method was used to select the most stable RG. A low average of SD value represented a low expression variance, or high stability. The dCt general analysis of both groups revealed the follow ranking order: *rpl13a* (2.26) > *eef1a1* (2.31) > *rpl7* (2.37) = *rplp2* (2.37) > *actb2* (2.46) > *actb1* (2.56) > *b2m* (2.88) > *eif2s2* (2.90) > *tuba1* (4.62) > *gapdh* (5.07) > *rps18* (5.11) (Fig. 2b). The analysis revealed the following order of stability of the evaluated genes from the non-transgenic group, from highest to lowest, without discriminating the tissues analyzed: *rpl13a* (2.09) > *eef1a1* (2.17) > *rplp2* (2.24) > *rpl7* (2.28) > *actb2* (2,30) > *actb1* (2.37) > *b2m* (2.46) > *eif2s2* (2.84) > *tuba1* (4.38) > *gapdh* (4.64) > *rps18* (4.76) (Supplementary Fig. 1a). The analysis revealed the following order of stability of the evaluated genes of the *gh*-transgenic group, from highest to lowest, without considering the tissues analyzed: *rpl13a* (2.43) > *eef1a1* (2.45) > *rpl7* (2.46) > *rplp2* (2.47) > *actb2* (2.61) > *actb1* (2.76) > *eif2s2* (2.98) > *b2m* (3.28) > *tuba1* (4.91) > *rps18* (5.44) > *gapdh* (5.46) (Supplementary Fig. 1b).

### GeNorm analysis

The geNorm analysis was carried out for the resulting data set following transformation of the Ct values into relative quantities through the 2^(minimum Ct value in a set sample − Ct value of a sample)^ formula and compared pairwise variation (SD values) for each gene pair. Then, the geometric mean of SD values was used to calculate the M-value. The generation of low average expression stability represents a low variance. The geNorm general ranking order for both groups was: *rpl13a*/*rpl7* (0.65) > *eef1a1* (0.68) > *rplp2* (0.89) > *actb2* (1.16) > *actb1* (1.26) > *b2m* (1.42) > *eif2s2* (1.64) > *tuba1* (2.17) > *gapdh* (2.74) > *rps18* (3.17) (Fig. 2c). In the non-transgenic group, the stability sequence of the genes evaluated independently of the tissue, from highest to lowest was: *rpl13a*/*eef1a1* (0.75) > *rpl7* (0.76) > *rplp2* (0.91) > *actb2* (1.14) > *actb1* (1.21) > *b2m* (1.28) > *eif2s2* (1.54) > *tuba1* (2.06) > *gapdh* (2.56) > *rps18* (2.96) (Supplementary Fig. 2a). The geNorm analysis revealed the following order of stability classification of genes in the *gh*-transgenic group, independent of tissue: *rpl13a*/*rpl7* (0.52) > *eef1a1* (0.59) > *rplp2* (0.85) > *actb2* (1.16) > *actb1* (1.30) *eif2s2* (1.54) > *b2m* (1.73) > *tuba1* (2.30) > *rps18* (2.93) > *gapdh* (3.39) (Supplementary Fig. 2b).

### NormFinder analysis

NormFinder was applied to analyze the most stable evaluated genes. A low mean expression stability represents a low variance. The general consensus result for both groups generated by NormFinder was: *rpl13a* (0.42) > *eef1a1* (0.80) > *rplp2* (0.88) > *rpl7* (0.94) > *actb2* (1.07) > *actb1* (1.28) > *eif2s2* (1.59) > *b2m* (1.88) > *tuba1* (4.18) > *rps18* (4.61) > *gapdh* (4.65) (Fig. 2d). The NormFinder analysis revealed that the order of stability classification in the non-transgenic group independent of tissue was: *rpl13a* (0.38) > *eef1a1* (0.76) > *rplp2* (0.96) > *actb2* (1.00) > *rpl7* (1.07) > *actb1* (1.12) > *b2m* (1.34) > *eif2s2* (1.75) > *tuba1* (3.97) > *gapdh* (4.25) > *rps18* (4.31) (Supplementary Fig. 3a). In *gh*-transgenic, the stability sequence of the genes evaluated independently of the tissues from the highest to the lowest was: *rpl13a* (0.65) > *rplp2* (0.72) > *rpl7* (0.80) > *eef1a1* (0.87) > *actb2* (1.15) > *actb1* (1.44) > *eif2s2* (1.46) > *b2m* (2.33) > *tuba1* (4.45) > *rps18* (4.91) > *gapdh* (5.02) (Supplementary Fig. 3b).

### BestKeeper analysis

The BestKeeper analysis provided two-interpretation-ways to rank the gene stability: one based on the samples SD values of Ct and other based on the Pearson’s correlation of expression among the genes. Thus, the genes with low SDs and high correlation with the BestKeeper index (indicating high similarity among the expression levels of the RGs) are ranked as the most stable genes. The SD values were used to reach a consensus regarding the comprehensive ranking, which is a more conservative approach. However, to construct the rankings of the most stable genes with BestKeeper, [r] and P values form the Pearson’s correlation were employed. This method is more sophisticated and statistically robust, as it results in rankings more similar to the ones obtained using other algorithms. The BestKeeper general analysis based on Pearson's correlation and combining tissues and both non-transgenic and *gh*-transgenic groups revealed the follow ranking order of best candidate RGs: *rpl13a* (0.90) > *eif2s2* (0.88) > *eef1a1* (0.85) > *gapdh* (0.85) > *rplp2* (0.83) > *rpl7* (0.83) > *actb2* (0.81) > *actb1* (0.76) > *rps18* (0.74) > *b2m* (0.63) *tuba1* (0.01) (Fig. 2e). The analyses revealed the following order of stability classification in the non-transgenic group independent of tissue: *rpl13a* (0.93) > *eif2s2* (0.90) > *eef1a1* (0.87) > *actb2* (0.85) > *actb1* (0.83) > *rplp2* (0.83) > *rpl7* (0.81) > *gapdh* (0.79) > *b2m* (0.78) > *rps18* (0.78) > *tuba1* (0.02) (Supplementary Fig. 4a). In *gh*-transgenics, the stability sequence of the genes evaluated independently of the tissues: *gapdh* (0.91) > *rpl13a* (0.87) > *eif2s2* (0.86) > *rplp2* (0.85) > *rpl7* (0.84) > *eef1a1* (0.82) > *actb2* (0.76) > *rps18* (0.75) > *actb1* (0.66) > *b2m* (0.47) > *tuba1* (0.001) (Supplementary Fig. 4b).

### Pairwise variation

**Figure 4:**
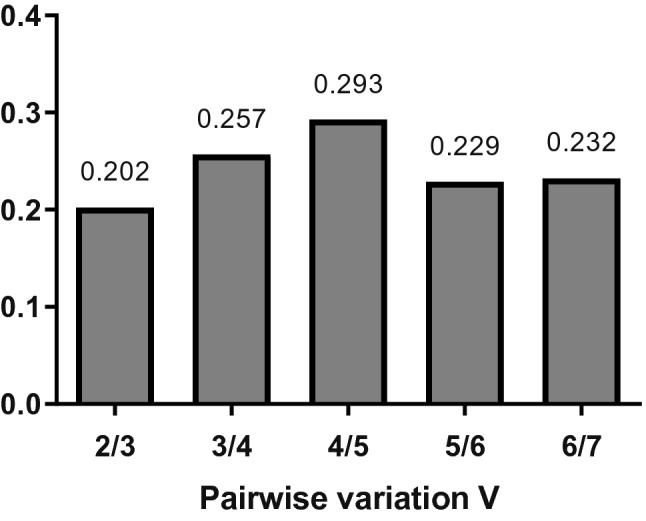
Graphical representation of the pairwise variation analysis. Y-axis represents V value and X-axis represents the number of reference genes used to normalize the reaction. The lowest value is found for 2/3 reference genes, what means that the addition of a third reference gene is superfluous to obtaining reliable results in experiments using non-transgenic and *gh*-transgenic zebrafish (*Danio rerio*) of F0104 lineage.

The analysis of average pairwise variations showed that the lowest V value was between 2 and 3 RG (V2/V3 = 0.202) indicating that the inclusion of a third RG would not be necessary to normalize gene expression. All the other V values resulting of the analysis were higher than the V value of 0.202, indicating that the inclusion of a third, fourth, fifth, sixth, or seventh RG would be superfluous to obtaining reliable results (Fig. 4).

In the present study, V values for the tissues and both non- and *gh*-transgenic groups combined were higher than the threshold value of 0.15 described by Vandesompele et al. (2002)^24^. The manual of the geNorm software suggests that the 0.15 value must not be taken as a strict cut-off and may vary according to each experimental design. Therefore, it is proposed that two of the best candidate RG should be used to obtain accurate and reliable results using the different tissues of the *gh*-transgenic zebrafish and its non-transgenic counterpart from the F0104 lineage.

### Verification

The verification was made using two selected RGs, as this number has been the best established by the average pairwise variation analysis. Considering the bests RGs of all grouped tissues, *rpl13a* and *rpl7* were selected for qRT-PCR data normalization. To analyze the tissues separately, the results found for each tissue were considered: for brain and intestine, *rpl13a* and *eef1a1*; for muscle, *rpl13a* and *actb2*.

**Figure 5:**
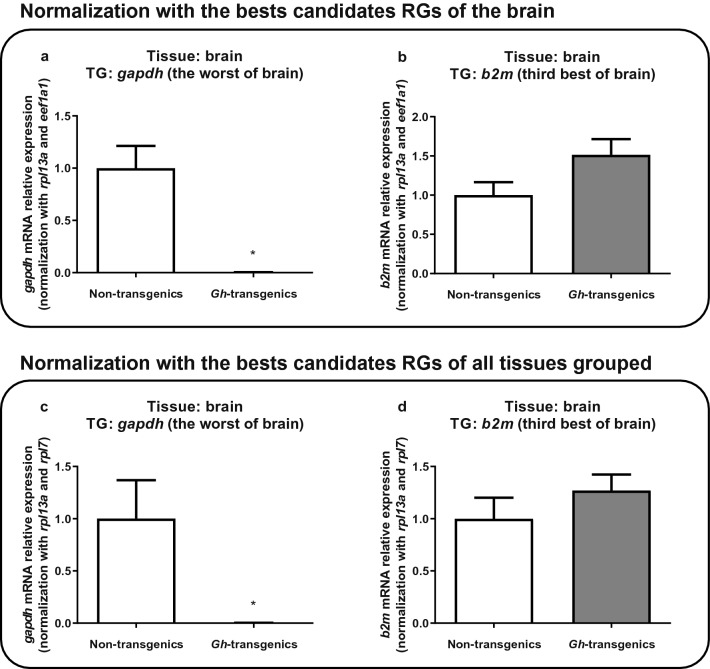
Verification of the suitability of the reaction when normalized with the candidate reference genes ranked as the best for brain tissue (**a**,**b**) and for all tissues grouped (**c**,**d**), using brain gene expression results. As target genes, it was chosen the worst (**a**) and the third best (**b**) for brain. In all the graphics, the Y-axis represents mRNA relative expression, and X-axis indicates the experimental group: non-transgenic and *gh*-transgenic zebrafish of F0104 lineage. Data are expressed as mean ± standard error of the mean. Asterisk indicate significant difference mean values between groups (n = 21; p < 0.05).

For the brain analysis, *gapdh* was chosen to be tested as a target gene, as it was found to be the less stable RG in this tissue, while *b2m* was choosen as a suitable RG, as it was the third best in previous analyses (stressing that the two most stable genes were used to normalize the test) (Fig. 5). It is possible to observe that even considering only the dataset taken the brain as for all tissues grouped, *gapdh* presented a high variation of gene expression between non-transgenic and *gh*-transgenic groups, being significantly more expressive (p < 0.05) in the *gh*-transgenic group (Fig. 5a,b), while for *b2m*, this expression did not present statistical variation (p > 0.05) between the groups (Fig. 5c,d).

**Figure 6:**
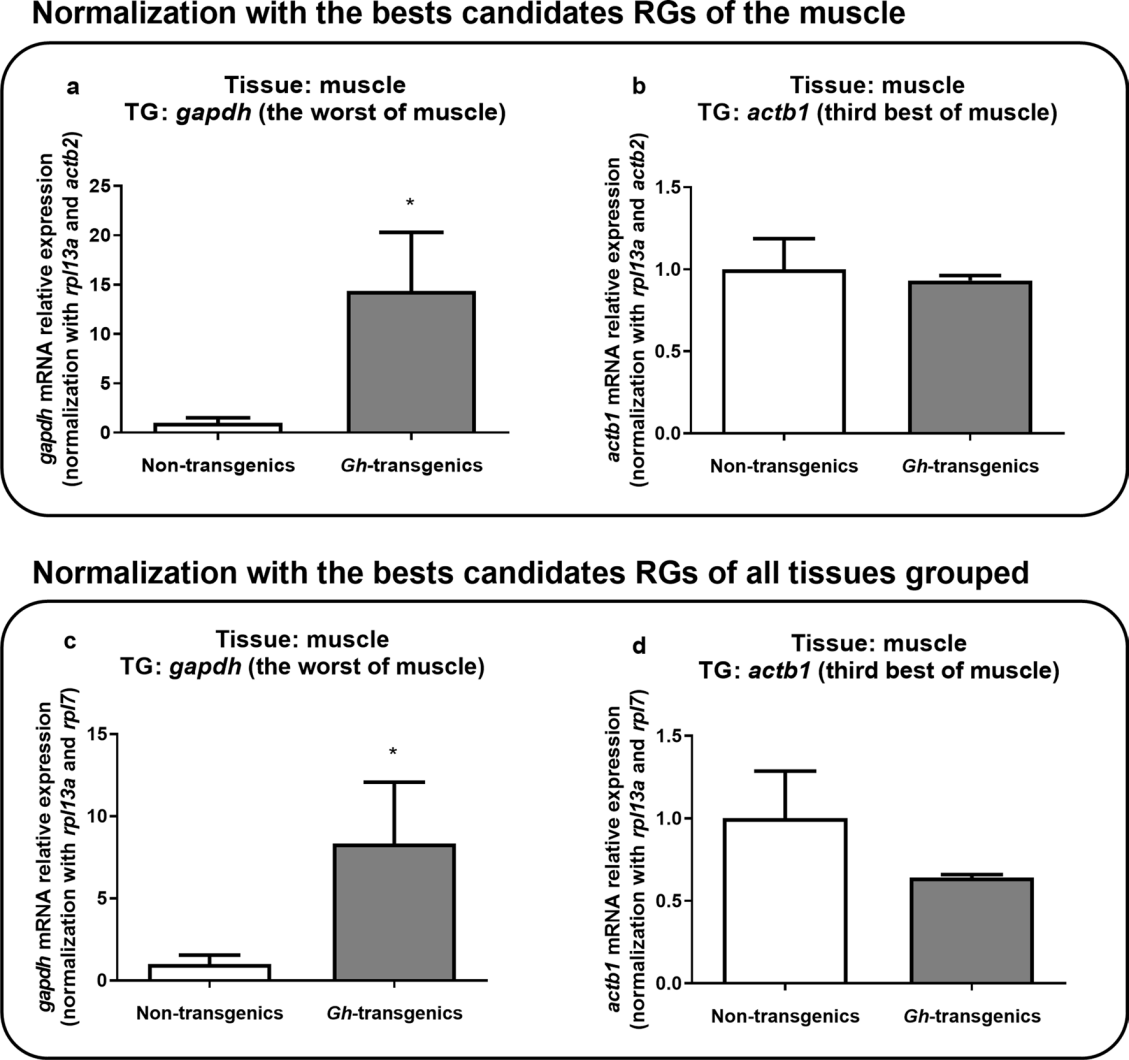
Verification of the suitability of the reaction when normalized with the candidate reference genes ranked as the best for muscle tissue (**a**,**b**) and for all tissues grouped (**c**,**d**), using muscle gene expression results. As target genes, it was chosen the worst (**a**) and the third best (**b**) for muscle. In all the graphics, the Y-axis represents mRNA relative expression, and X-axis indicates the experimental group: non-transgenic and *gh*-transgenic zebrafish of F0104 lineage. Data are expressed as mean ± standard error of the mean. Asterisk indicate significant difference mean values between groups (n = 7; p < 0.05).

A similar pattern was found in the muscle analysis, in which the target genes used for verification were *gapdh* (the worst) and *actb1* (the third best) (Fig. 6). *Gapdh* expression was not stable between non-transgenic and *gh*-transgenic groups, even for muscle (Fig. 6a), as for all grouped tissues (Fig. 6c), being statistically higher in the *gh*-transgenic group (p < 0.05). Incidentally, *actb1* did not vary (p > 0.05) between the groups and the different analysis (Fig. 6b,d).

**Figure 7:**
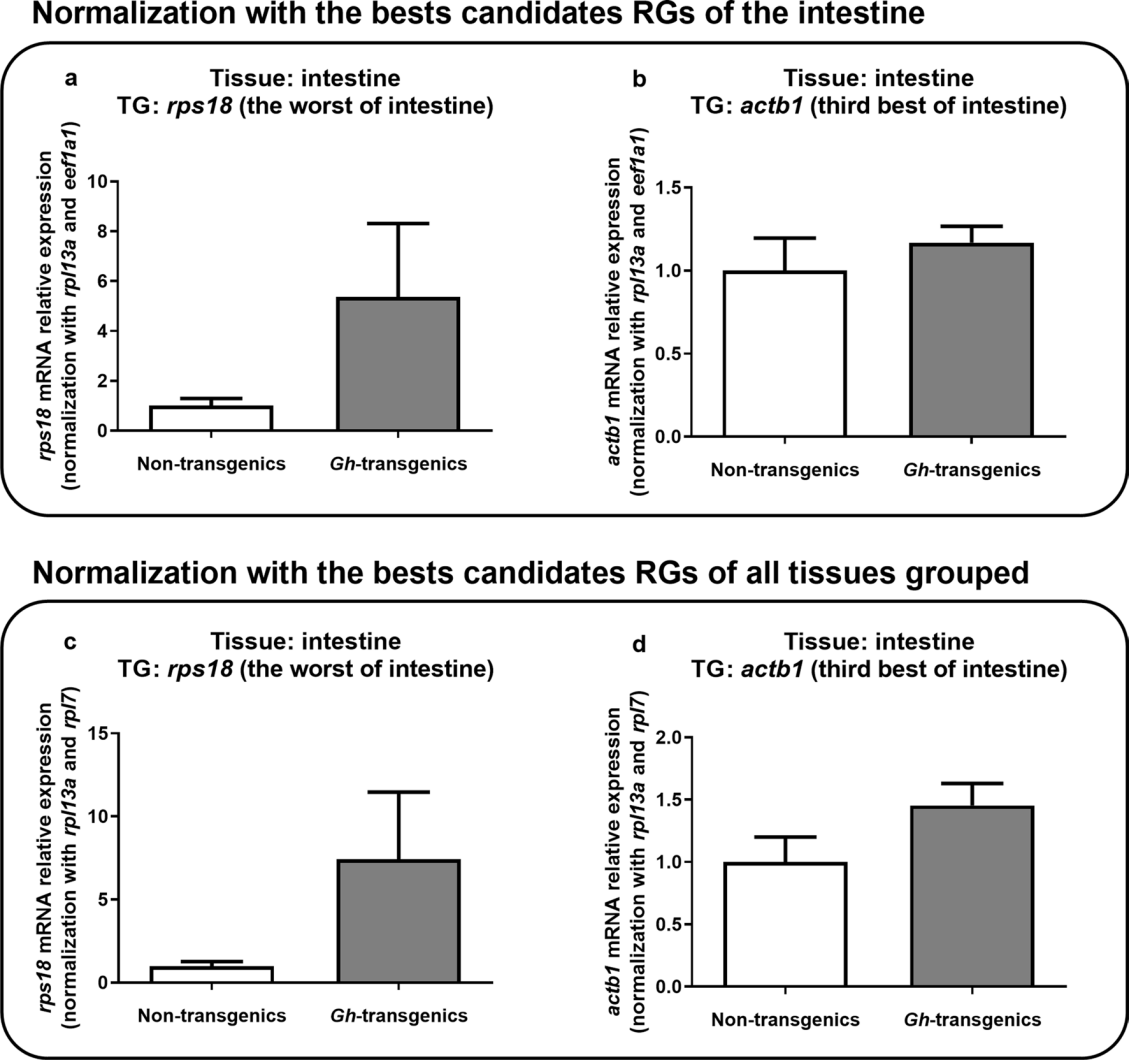
Verification of the suitability of the reaction when normalized with the candidate reference genes ranked as the best for intestine tissue (**a**,**b**) and for all tissues grouped (**c**,**d**), using intestine gene expression results. As target genes, it was chosen the worst (**a**) and the third best (**b**) for intestine. In all the graphics, the Y-axis represents mRNA relative expression, and X-axis indicates the experimental group: non-transgenic and *gh*-transgenic zebrafish of F0104 lineage. Data are expressed as mean ± standard error of the mean. Asterisk indicate significant difference mean values between groups (n = 7; p < 0.05).

Regarding the intestine, the target genes chosen were *rps18* (the worst) and *actb1* (third best) (Fig. 7). In this analysis, both genes we found to not vary between groups (p > 0.05), considering the results of only this tissue (Fig. 7a,b) as for all grouped tissues (Fig. 7c,d).

**Figure 8:**
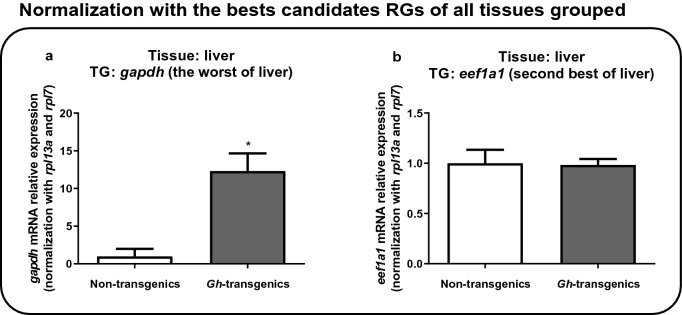
Verification of the suitability of the reaction when normalized with the candidate reference genes ranked as the best for all tissues grouped (**a**,**b**), using liver gene expression results. As target genes, it was chosen the worst (**a**) and the second best (**b**) for liver. In the two graphics, the Y-axis represents mRNA relative expression, and X-axis indicates the experimental group: non-transgenic and *gh*-transgenic zebrafish of F0104 lineage. Data are expressed as mean ± standard error of the mean. Asterisk indicate significant difference mean values between groups (n = 7; p < 0.05).

As for the liver, the target genes selected were *gapdh* (the worst) and *eef1a1* (second best) (Fig. 8). The *gapdh* gene was significantly overexpressed (p < 0.05) in the *gh*-transgenic group (Fig. 8a), demonstrating its instability, which had already been verified by the stability analysis. On the other hand, the *eef1a1* gene presents expressions levels without significant difference (p > 0.05) between the non-transgenic and *gh-*transgenic groups (Fig. 8b).

## Discussion

In this study, we present the most extensive evaluation of candidates for normalizing genes for qRT-PCR in *gh*-transgenic and non-transgenic zebrafish groups. In both groups, we evaluated 11 genes in 4 different tissues, and the results were analyzed using 5 different bioinformatic approaches. In addition, consensus analyses were performed between the organs and between the groups.

F0104 is a zebrafish transgenic lineage, developed in 2004, that overexpresses the GH from silverside (*Odontesthes argentinensis*) in a constitutive promoter (*actb*), and also carries the gene of green fluorescent protein (GFP) as a marker^17^. This lineage has been very important as an animal model, not only for elucidating the effect of GH overexpression in tissues and its effect in different physiologic systems^17–19, 21, 29–33^, but also to evaluate the interaction of this hormone with other proteins in the organism^34–37^. To achieve all those elucidations, quantitative PCR analysis has been applied in diverse studies using this lineage. Different RGs have been used to normalize and validate the results, but the most widely used was *eef1a1*^18, 19, 21, 30–32, 35–38^. As *eef1a1* was the third most stable RG found here (Fig. 2a) for the same lineage, those previous results corroborate our data. In general, our analyses found the *eef1a1* gene to be highly stable, occupying between the first and at least the third position in the rankings—except in the BestKeeper and NormFinder analysis in the *gh*-transgenic group—demonstrating to be one of the most specific and stable gene for analysis in zebrafish in both groups. Other studies have also examined the stability of RGs in non-transgenic zebrafish, showing that *eef1a1* is a stable gene for qRT-PCR normalization^11–13, 15, 16^.

However, *eef1a1* was not ranked as the most stable gene among all analyzed in this study. Here, we could verify that in a RefFinder consensus analysis between groups and tissues, the most stable gene was *rpl13α*, followed by *rpl7* (Fig. 2)*.* However, when the consensus analysis was verified considering the groups and the tissues individually, the positions in stability rankings can be altered (Tables 2, 3, 4; Supplementary Figs. 1–4). In this case, *rpl13α* was not the most stable in all the tissues from the different groups, but only in the intestine in the non-transgenic group (Table 3). The *rpl7* gene, which occupied the second position in the consensus of general stability, was shown to be the third most stable in the liver of the non-transgenic group (Table 3), and in the muscle of the *gh-*transgenic group (Table 4). The high stability of *rpl13α* in zebrafish liver has also been demonstrated by Xu et al. (2013)^39^ when fish were stressed with arsenate. The *rpl13α* was also shown to be a more stable and reliable gene for chemical treatments in a recent study using zebrafish^1^, consistent with previous studies^11, 40, 41^.

Considering the analysis by tissues, some studies corroborate our findings. Jaramillo et al. (2018)^16^ found that the best RGs in the brain were *rpl8* and *actb1*; the former was not investigated in our study, but the latter was also found by us as the best for that tissue in the non-transgenic group (Table 3), and the fourth best in the *gh*-transgenic group (Table 4). The results obtained by Lang et al. (2016)^15^ were compatible with our findings, where *rpl13α, rpl7, actb2* and *eef1a1* were the most suitable RGs in liver when exposed in different concentration groups to cadmium and subsequently infected with *Aeromonas hydrophila*. The same study found that in the intestine the best RGs were *ef1a1, rnf7*, *rplp2* and *rpl13α. *In our study, we did not investigate *rnf7*, and the other genes appeared in different ranking positions: *rpl13α* was the best RG for intestine in non-transgenic zebrafish; *ef1a1* was at 5th and *rplp2* was at 8th place (Table 3).

*Gapdh* and *rps18* showed to be the most inappropriate genes for both groups in terms of stability—with the exception of the analysis carried out using the algorithm BestKeeper, where surprisingly *gapdh* occupied the first place in the *gh*-transgenic group when considering all tissues together (Supplementary Fig. 4). In general, both *gapdh* and *rps18,* when analyzed separately in the tissues, occupied the last positions in almost all analyses and statistical programs. This result demonstrates that they are unsuitable RGs, and when they are used, they can seriously compromise the analysis and the accuracy of qRT-PCR results. Also considering the rankings generated by our analysis, *tuba1* was the third less stable gene in both groups separately (Fig. 3) and together in most cases (Fig. 2a–d). For wild zebrafish, *gapdh*^11, 12^ and *tuba1*^12^ have already been reported as unstable genes and unsuitable RG.

Another result found in our study was that the use of two RGs to interpret qRT-PCR results was enough (Fig. 4). This result is positive, as needing less genes to evaluate the analysis makes it cheaper and simpler. Furthermore, this result reinforces the methods and results obtained in previous studies with *gh*-transgenic zebrafish, which also used two RGs for qRT-PCR analyses^30, 31, 33, 36–38^. Some authors of these previous studies also analyzed candidate RGs with computational algorithms (i.e. geNorm)^31, 32, 36, 38^. However, this is the first study to use an integrative approach with various methods to evaluate the variability of candidate RGs.

The verification analysis, which was made for each tissue, considering the best and worst candidate RGs found as target genes by each tissue and for all grouped tissues, has also shown the difference of gene expression between suitable and unsuitable RGs, highlighting the risk of choosing few and unstable genes to normalize the dataset. Besides it was possible to test if each tissue needs to be evaluated individually or if the grouped results are enough to access the bests RGs to use in a specific experiment with the F0104 lineage (Figs. 5, 6, 7). The second situation (grouped results) can make an experiment quicker and less costly because fewer RGs are needed to obtain reliable results. The results from brain, muscle, and liver clearly show the difference between a suitable and unsuitable (*gapdh*) RG, showing a stable expression of the second/third best RG and variable expression of the *gapdh* RG (Figs. 5, 6, 8). In the intestine, even though the gene expression did not presenting a statistic difference between the groups, a stronger trend of variability was observed in *rps18* expression in both analysis, when compared to the *actb1* results (Fig. 7).

Yet, the verification method revealed that the *gapdh* is overexpressed in muscle and liver (Figs. 6, 8), while it is downregulated in the brain of *gh*-transgenic zebrafishes (Fig. 5). This gene encodes to an enzyme with diverse function for maintaining cell homeostasis, but its main function is to break down glucose in the glycolysis. The GH hormone induces IGF-1 production and also promotes postnatal body growth^42^, which explains the impair of glycolysis in the liver and muscle of fish in the *gh*-transgenic group. Maybe, the downregulation of *gapdh* in the brain can be explained by the downregulation of glucose transport-related genes in the brain of fish form the F0104 lineage^37^.

Furthermore, the maintenance of the response pattern of target gene expression independently of the set of genes (or the two best RGs of specific tissue or the two best consensual RGs after tissue grouping) selected to normalize the qRT-PCR (see in Figs. 5, 6, 7, 8) indicates that a unique consensual set of genes can be used to normalize the data of various tissues. Hence, instead of selecting two RGs for the brain, two RGs for the intestine, two RGs for the liver, and two RGs for the muscle, the researcher could select only two RGs to normalize the results of qRT-PCR for these four different tissues.

Regarding the differences in stability of the candidate genes, probably this result happened due to the different functions and heterogeneity of the organs^43^. Besides that, the different results presented by each algorithm are expected due to the distinct statistical approach that was used to construct the rankings^44^. The discrepancies in the ranks of the most stable genes obtained by each software reflect the differences of the algorithms and procedures of each method and demonstrate the need to evaluate the RGs using several bioinformatic algorithms^16, 23^.

Therefore, it is important to consider that RGs for qRT-PCR can be altered by different experimental conditions, organs, gender, stage of development and chemical concentration^11, 16, 24^. So, it is impossible to find suitable RGs that exhibit constant expression pattern for all species and under all experimental conditions^6, 45^. According to the Minimum Information for Publication of Quantitative Real Time PCR Experiments (MIQE)^46^, a RG needs to be validated and established for each species and for different physiological conditions. Our results show that although the literature has already demonstrated alterations in the metabolism of the F0104 lineage^19, 21, 29–35, 38, 47, 48^, these animals, in comparison to non-transgenic animals, did not suffer notable variations in the expression of most of the analyzed genes.

In summary, for the first time, RGs for non-transgenic and *gh*-transgenic zebrafish of F0104 lineage were evaluated for gene expression stability and these results support the use of two stable RGs for proper interpretation of the qRT-PCR results among different tissues collected in specific experiments (brain, instestine, liver, and muscle in this case). The *actb1* and *actb2* genes were shown to be more stable and suitable for intestine and muscle, while in brain and liver, the *eef1a1* gene was shown to be the most suitable. In addition to the rankings of the most suitable candidate RGs in each tissue, we presented a consensual analysis that showed that the genes *rpl13α*, *rpl7* and *eef1a1* were the most stable between tissues and groups. More than 30 studies have already been performed using zebrafish of the F0104 lineage and our conclusions can be used in countless future studies which analyze the results of qRT-PCR in this lineage, the gold standard technique to evaluate gene expression in experimental samples. In addition, the present study showed the capital importance of using various methods to evaluate stability. As seen in our case, if used individually, BestKeeper could have led to a complete misinterpretation, pointing *gapdh* as the most stable gene in the *gh*-transgenic group considering all tissues together. Moreover, the risks of a mistaken choice of RG and its consequences for the interpretation of results were demonstrated visually through the verification steps. Finally, this study allowed us to conclude that genes that are commonlly used in mammals for qRT-PCR assays have low stability in both non-transgenic and *gh*-transgenic zebrafish. This reinforces the importance of using species-specific genes, instead of ‘consecrated’ RGs for normalization and, wherever possible, using RGs that have been previously checked for stability based on the animal model used and the specific experimental conditions. Therefore, in a near future more screenings studies about RGs in other genetically engineered lineages will be needed to guarantee reliable interpretations about the expressions of the studied genes.

## Supplementary information

Supplementary figures.
