# Supplementary material for: Evaluation of qPCR reference genes in GH-overexpressing transgenic zebrafish (Danio rerio)
Source: Sci Rep. 2020 Jul 29;10:12692. doi: 10.1038/s41598-020-69423-y (PMC7391647; doi:10.1038/s41598-020-69423-y)
Supplement: Supplementary file 1 — Supplementary figures. [file 41598_2020_69423_MOESM1_ESM.docx]

**Evaluation of qPCR reference genes in GH-overexpressing transgenic zebrafish**

**(*Danio rerio*)**

Gabriela T. Rassier^1^, Tony Silveira^4^, Mariana H. Remião^2^, Larissa O. Daneluz^2^, Amanda W. S. Martins^2^, Eduardo N. Dellagostin^2,^ Hadassa G. Ortiz^2^, William B. Domingues^2^, Eliza R. Komninou^3^, Mateus T. Kütter^4^, Luis F. Marins^4^, Vinicius F. Campos^1,2*^

^1^Programa de Pós-Graduação em Bioquímica e Bioprospecção, Centro de Ciências Químicas, Farmacológicas e de Alimentos. Universidade Federal de Pelotas, Pelotas, RS, Brasil.

^2^Laboratório de Genômica Estrutural, Programa de Pós-Graduação em Biotecnologia, Centro de Desenvolvimento Tecnológico, Universidade Federal de Pelotas, Pelotas, RS, Brasil.

^3^Laboratório de Reprodução Animal, Programa de Pós-Graduação em Veterinária, Universidade Federal de Pelotas, Pelotas, RS, Brasil.

^4^Laboratório de Biologia Molecular, Instituto de Ciências Biológicas, Universidade Federal do Rio Grande, Rio Grande, RS, Brasil.

* Corresponding author: Vinicius Farias Campos - Biotecnologia, Universidade Federal de Pelotas, Campus Universitário s/n, Pelotas, RS, Brazil, Cep: 96010-900. RS, Brazil; Phone number: +555332757350; E-mail: [fariascampos@gmail.com](mailto:fariascampos@gmail.com)


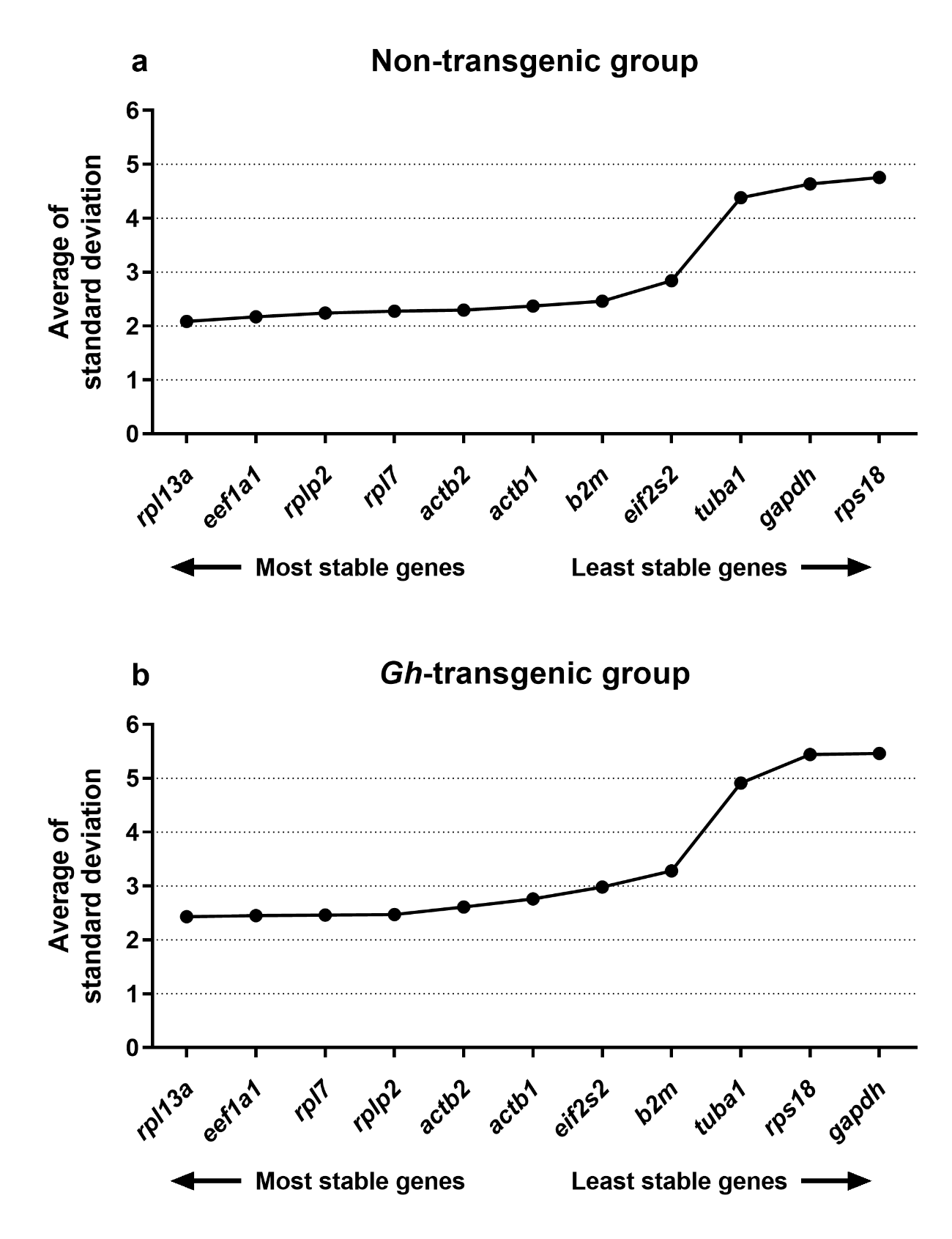


Supplementary Fig. 1. Stability analysis of the candidates reference genes in non-transgenic and transgenic zebrafish (*Danio rerio*) of F0104 strain calculated by comparative delta Ct (dCt) method. General gene expression stability in non-transgenic zebrafish (a) and in *gh*-transgenic zebrafish (b). Data are expressed as average of standard deviation. The most stable genes are displayed on the left, and the least stable genes are displayed on the right of the x-axis.


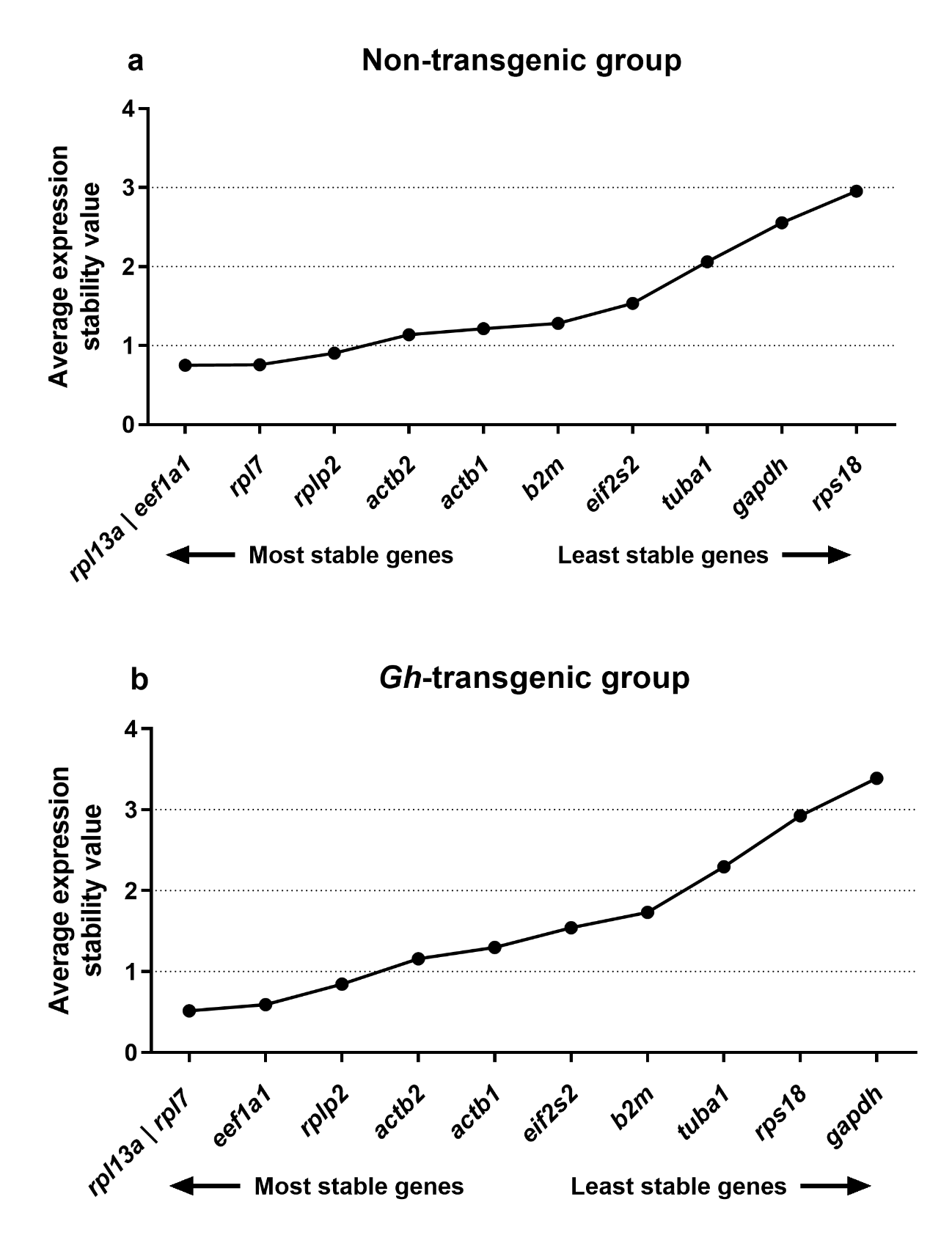


Supplementary Fig. 2. Stability analysis of the candidates reference genes in non-transgenic and transgenic zebrafish (*Danio rerio*) of F0104 strain calculated by geNorm algorithm. General gene expression stability in non-transgenic zebrafish (a) and in *gh*-transgenic zebrafish (b). Data are expressed as average expression stability values. The most stable genes are displayed on the left, and the least stable genes are displayed on the right of the x-axis.


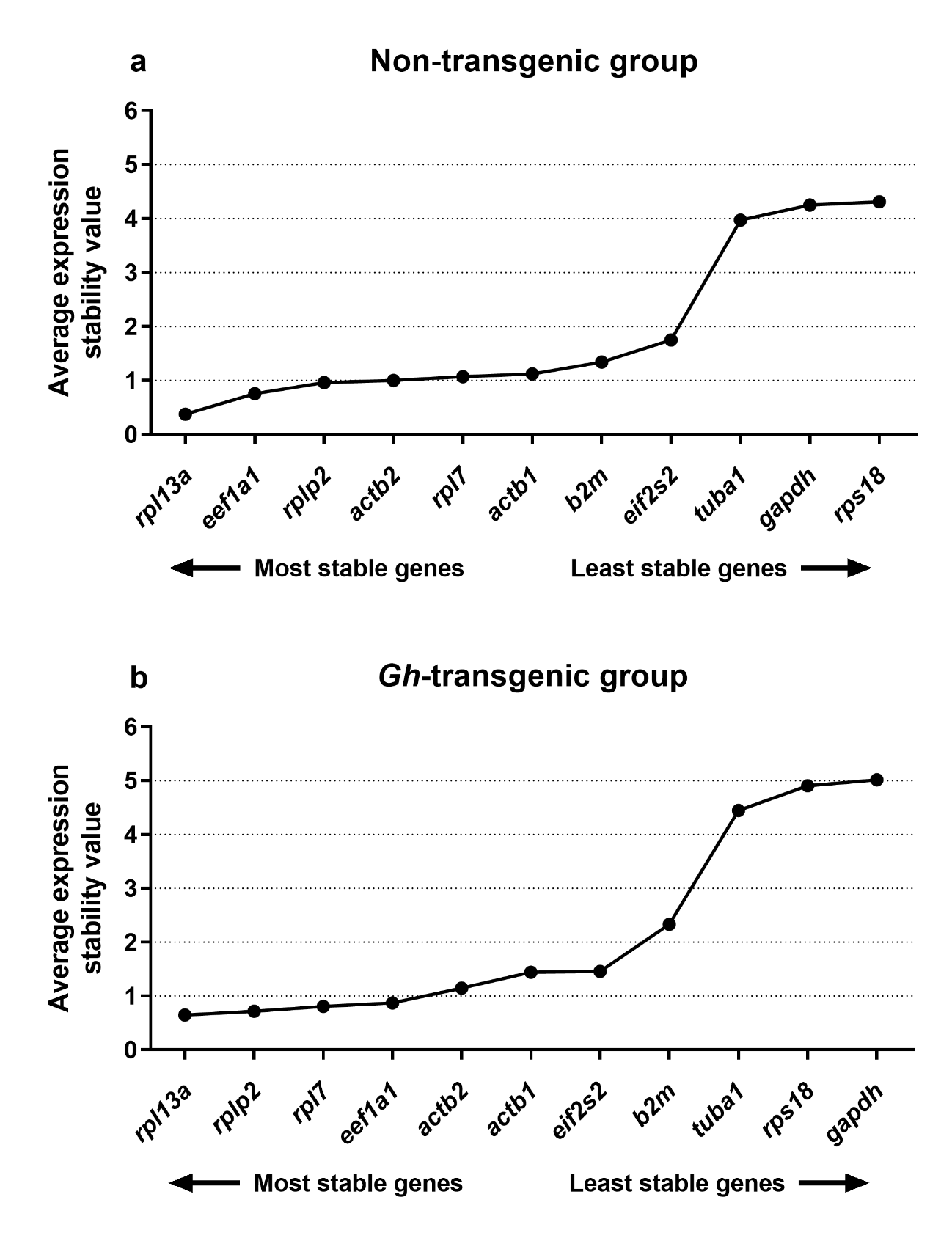


Supplementary Fig. 3. Stability analysis of the candidates reference genes in non-transgenic and transgenic zebrafish (*Danio rerio*) of F0104 strain calculated by NormFinder algorithm. General gene expression stability in non-transgenic zebrafish (a) and in *gh*-transgenic zebrafish (b). Data are expressed as average expression stability values. The most stable genes are displayed on the left, and the least stable genes are displayed on the right of the x-axis.


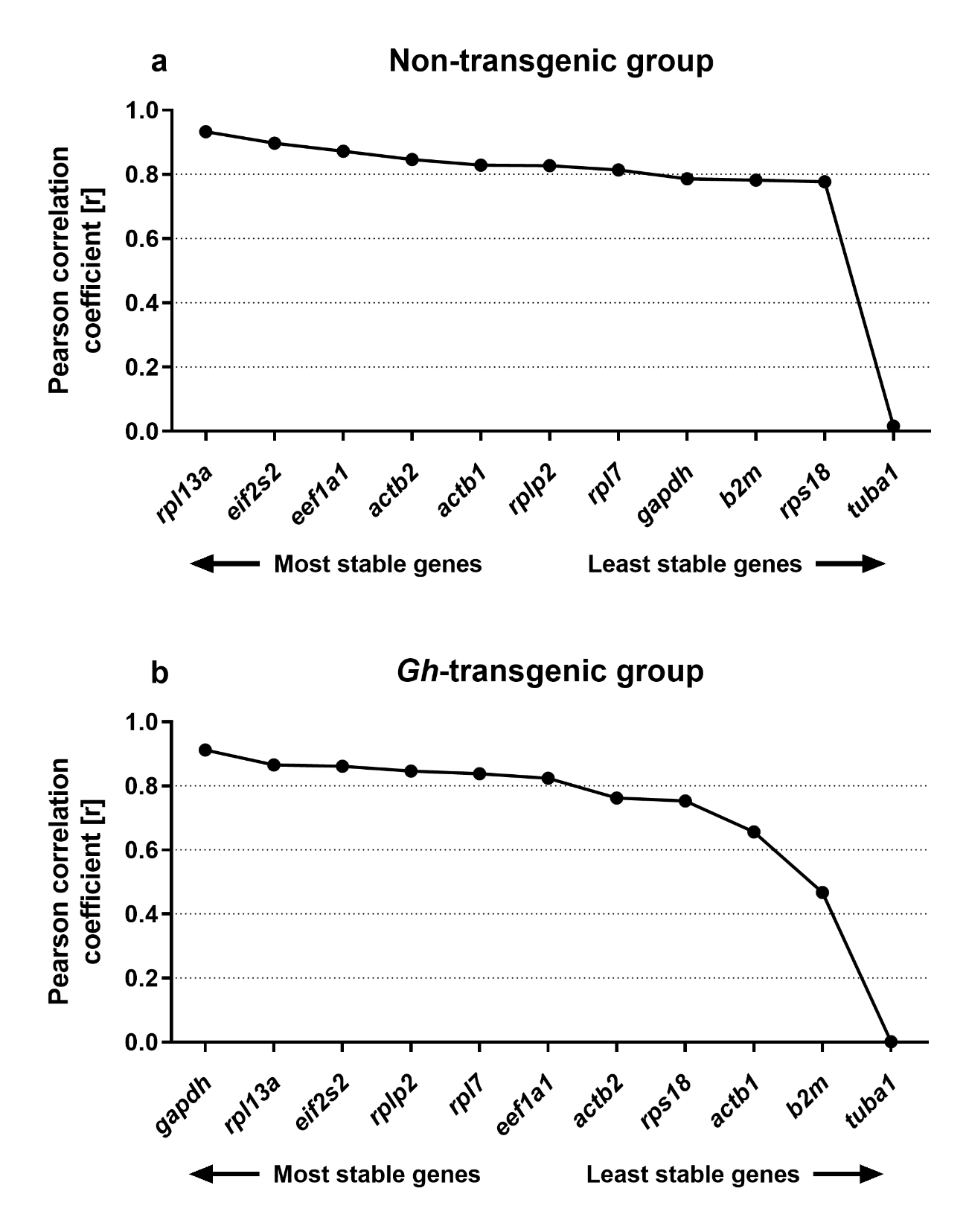


Supplementary Fig. 4. Stability analysis of the candidates reference genes in non-transgenic and transgenic zebrafish (*Danio rerio*) of F0104 strain calculated by BestKeeper algorithm. General gene expression stability in non-transgenic zebrafish (a) and in *gh*-transgenic zebrafish (b). Data are expressed as Pearson’s correlation coefficient. The most stable genes are displayed on the left, and the least stable genes are displayed on the right of the x-axis.
